# Combination of Pulmonary Tractotomy and Free Subcutaneous Fat Pad Coverage for Iatrogenic Lung Injury Caused by Chest Tube Insertion

**DOI:** 10.7759/cureus.56798

**Published:** 2024-03-23

**Authors:** Eito Niman, Kenji Takahashi

**Affiliations:** 1 Department of General Thoracic Surgery, National Hospital Organization Fukuyama Medical Center, Fukuyama, JPN

**Keywords:** iatrogenic lung injury, penetrating lung injury, chest tube, free subcutaneous fat pad, pulmonary tractotomy

## Abstract

Iatrogenic lung injury caused by chest tube insertion is a potential complication that requires careful attention, and thoracic surgeons should be knowledgeable about the appropriate management strategies if complications arise. This report describes a successful procedure for treating an iatrogenic lung injury. An 80-year-old Japanese man with severe emphysema complaining of breathlessness was diagnosed with a right secondary pneumothorax. Computed tomography revealed moderate adhesions in the thoracic cavity. Chest tube drainage was performed. Lung expansion was insufficient and massive air leakage continued. Repeat computed tomography showed the chest tube inserted into the right upper lobe. Thus, pulmonary tractotomy followed by free fat pad coverage was performed to successfully treat the iatrogenic lung injury caused by chest tube insertion. Since no air leakage was observed postoperatively, the chest tube was removed on the third postoperative day. The patient was discharged after two weeks of rehabilitation. Pulmonary tractotomy combined with free subcutaneous fat pad coverage would be effective for repairing iatrogenic lung injuries in patients with severe emphysema.

## Introduction

This article was previously presented as a meeting abstract at the 40th Annual Meeting of the Japanese Association of Chest Surgery on July 13, 2023.

Chest tube insertion is a widely performed procedure for managing pneumothorax, hemothorax, pleural effusion, and empyema. Although the procedure is fairly simple, a risk of severe complications exists. Ball et al. reported on the complications associated with chest tube insertion. Iatrogenic lung injury caused by a chest tube occurred in three percent of cases. In addition, approximately half of the malpositioned chest tubes were not evident on the chest radiograph [1]. Apart from these, injury to the intercostal artery, heart, diaphragm, and esophagus, and migration to the abdominal cavity can occur. In Japan, the Pharmaceuticals and Medical Devices Agency has raised concerns regarding the risk of these complications [2]. Treatment for such injuries is varied. Some cases may be relieved by conservative therapy, while others may require surgery including anatomical lung resection. Pulmonary tractotomy (PT) is an effective procedure for penetrating lung injury, thereby avoiding anatomical lung resection. This report describes an effective combined procedure for treating an iatrogenic penetrating lung injury in a patient with severe emphysema.

## Case presentation

**Figure 1:**
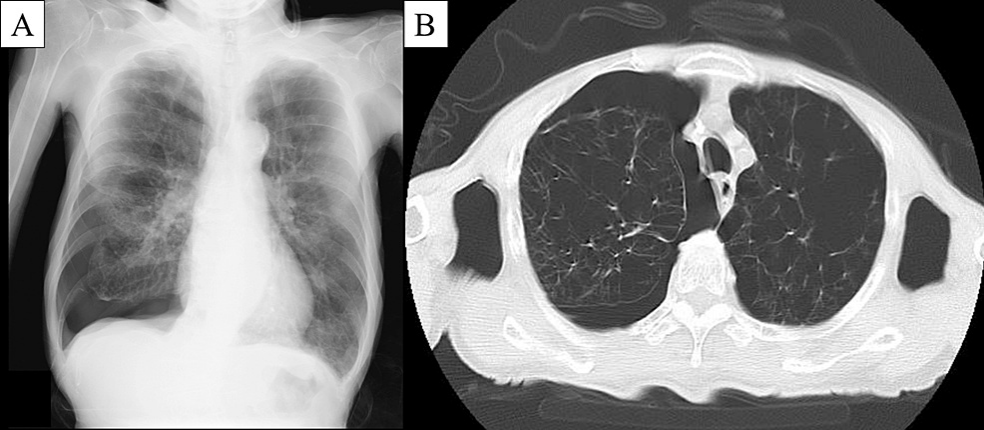
Chest radiography and computed tomography before chest tube insertion Chest radiography (A) and computed tomography (B) reveal a right pneumothorax in a patient with severe emphysema, and moderate adhesions between the lung and chest wall.

An 80-year-old Japanese man with severe emphysema presenting with breathlessness was diagnosed with a right secondary pneumothorax. The initial vital signs included a heart rate of 95 beats per minute, blood pressure of 109/68 mmHg, temperature of 36.1°C, and oxygen saturation of 96% under 3 L/min oxygen administration. The patient exhibited tachypnea and effort ventilation. Chest radiography and computed tomography (CT) revealed a collapsed lung and moderate adhesions in the thoracic cavity (Figure 1); however, sufficient space existed above the diaphragm to safely insert a chest tube.

**Figure 2:**
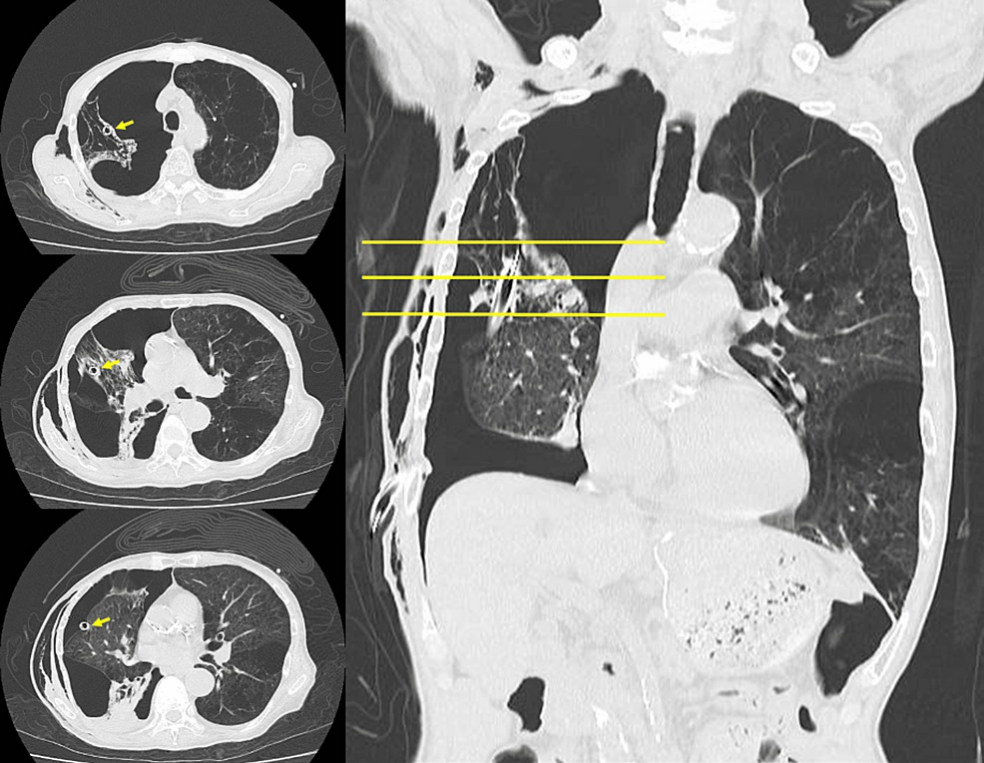
Computed tomography taken a day after chest tube insertion The chest tube was inserted 6 cm into the right upper lobe (yellow arrows), and lung collapse was worse than it was before the chest tube insertion. The images to the left of the coronal section are axial images at the level of the yellow lines.

Chest tube drainage was performed in the emergency room without encountering any resistance. A 24 Fr double-lumen chest tube was selected because of the possibility of later pleurodesis. Lung collapse was worse than it was before the procedure and massive air leaks continued. A repeat CT scan done the following day showed that the chest tube was inserted 6 cm into the right upper lobe (Figure 2), and massive subcutaneous emphysema had developed. It seemed difficult to control the air leak by chest tube drainage.

**Figure 3:**
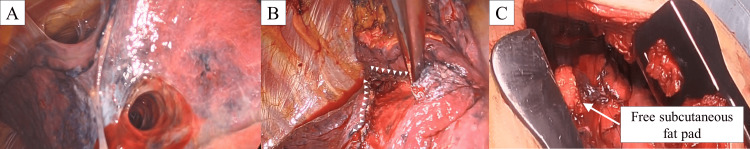
Intraoperative findings (A) The wound tract in the lung caused by chest tube insertion is easily identified. (B) Pulmonary tractotomy using a stapler opens the wound tract. The staple line is V-shaped (arrowheads). (C) A free subcutaneous fat pad, harvested from the thoracotomy wound, is placed on the open wound tract.

Therefore, urgent surgery was performed to repair the iatrogenic penetrating lung injury. The wound tract was identified easily by thoracoscopic exploration (Figure 3A); fortunately, no active bleeding was observed. A right thoracotomy through the fifth intercostal space with a 10 cm skin incision was done. After dissection of the adhesions between the right upper lobe and chest wall, the anvil of the stapler could be inserted into the wound tract smoothly and safely. PT using a stapler (Powered ECHELON FLEX® and GST® cartridge 60 mm green, Johnson & Johnson, USA) opened the wound tract (Figure 3B), and a massive air leak was observed during the water submersion test. A subcutaneous fat pad was harvested from the thoracotomy wound and placed on the open tract, together with fibrin glue (Figure 3C). To prevent tearing of the friable emphysematous lung, interrupted sutures through the staple line were placed to fix the free fat pad. The total duration of the operation was 98 minutes. No air leakage was observed postoperatively. The chest tube was removed on the third postoperative day. The patient was discharged after two weeks of rehabilitation.

**Figure 4:**
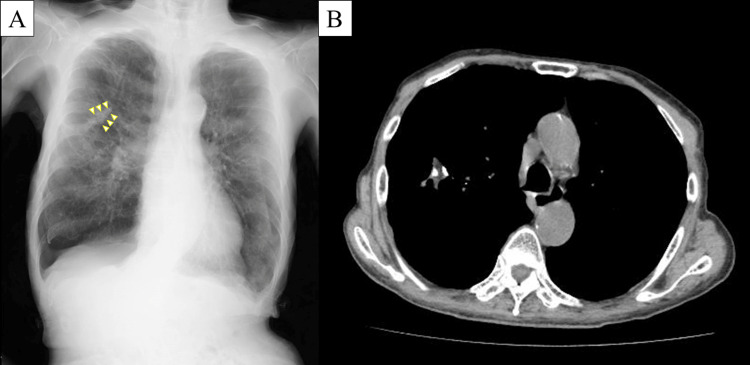
Chest radiograph and computed tomography taken at the time of right pneumothorax recurrence three months after surgery Chest radiograph (A) shows a V-shaped staple line (arrowheads), while computed tomography (B) shows the attached free fat pad had shrunk but remained.

Three months after surgery, the right pneumothorax recurred. Chest radiography and CT showed a V-shaped staple line, and the attached free fat pad had shrunk but remained intact (Figure 4). The pneumothorax was managed using chest tube drainage alone.

## Discussion

PT was first described by Wall et al. [3] in 1994 as a treatment for deep penetrating lung injury that does not involve the hilar vessels or airways. In 1997, Asensio et al. reported PT using a stapler [4]. PT is a simple and effective approach that allows direct control of bleeding and air leakage, with significant advantages such as shortened operation time and avoidance of anatomical lung resection. Although originally reported as a procedure for completely penetrating through-and-through lung injuries, Muraoka et al. [5] reported the safe performance of PT in lung injuries with no exit wounds by carefully inserting a stapler. However, caution must be exercised when blindly inserting a stapler into friable lung parenchyma, as there is a risk of secondary injury to the deep lung parenchyma, pulmonary vessels, or bronchioles. Oyamatsu et al. [6] reported a penetrating lung injury caused by a chest tube. The lung was fully penetrated intentionally, and the tract was used as a pathway for a Penrose drain to guide the stapler. In the present case, alternative repair procedures, such as direct suturing of the defect and filling the wound tract with fibrin glue or free subcutaneous fat pad, were considered, but they may leave a dead space and control of bleeding and air leakage seems uncertain. The emphysematous lung appeared too fragile to suture directly. Finally, PT was determined as the safest and most reliable repair procedure. Furthermore, the thickness of the 24 Fr double-lumen chest tube stabbing the lung parenchyma likely facilitated the insertion of the stapler into the wound tract. To control hemorrhage and air leakage in the open wound tract created by PT, direct suturing and use of an argon beam coagulator [7] have been reported. There have been no reports on the combined use of PT and free subcutaneous fat pad coverage for the treatment of penetrating lung injuries.

Controlling air leakage during pulmonary surgery remains challenging. Recently, coverage of damaged lung tissue with free subcutaneous or pericardial fat pad has been reported [8-10]. This technique is considered safe, easy, and effective because the period of chest drain placement is shortened compared to those in techniques involving fibrin glue and absorbable sheets [8,10]. Over half of the sutured free fat pads remain on CT images six months after surgery [8]. In patients who underwent additional pulmonary resection with sutured-free pericardial fat pads, fat necrosis surrounded by fibrous tissue was observed histologically, but the morphological integrity of the fat tissue was maintained. Fat tissue adhering to the pleural defect without angiogenesis develops into a thoracolithiasis-like condition [10,11]. In the present case, residual fat tissue was confirmed on CT images obtained when pneumothorax recurred three months after surgery.

## Conclusions

This report describes the successful repair of an iatrogenic penetrating lung injury caused by a chest tube. Although iatrogenic lung injury from chest tube insertion is rare, thoracic surgeons should be knowledgeable about the appropriate management strategies if complications occur. Both PT and free subcutaneous fat pad coverage are safe and easy. The combination of these procedures is an effective option for repairing penetrating lung injuries in patients with severe emphysema.
